# Quantifying the Effect of Ribosomal Density on mRNA Stability

**DOI:** 10.1371/journal.pone.0102308

**Published:** 2014-07-14

**Authors:** Shlomit Edri, Tamir Tuller

**Affiliations:** The Department of Biomedical Engineering, Tel-Aviv University, Tel-Aviv, Israel; Pohang University of Science and Technology, Korea, Republic of

## Abstract

Gene expression is a fundamental cellular process by which proteins are eventually synthesized based on the information coded in the genes. This process includes four major steps: transcription of the DNA segment corresponding to a gene to mRNA molecules, the degradation of the mRNA molecules, the translation of mRNA molecules to proteins by the ribosome and the degradation of the proteins. We present an innovative quantitative study of the interaction between the gene translation stage and the mRNA degradation stage using large scale genomic data of *S. cerevisiae*, which include measurements of mRNA levels, mRNA half-lives, ribosomal densities and protein abundances, for thousands of genes. The reported results support the conjecture that transcripts with higher ribosomal density, which is related to the translation stage, tend to have elevated half-lives, and we suggest a novel quantitative estimation of the strength of this relation. Specifically, we show that on average, an increase of *78%* in ribosomal density yields an increase of *25%* in mRNA half-life, and that this relation between ribosomal density and mRNA half-life is not function specific. In addition, our analyses demonstrate that ribosomal density along the entire ORF, and not in specific locations, has a significant effect on the transcript half-life. Finally, we show that the reported relation cannot be explained by different expression levels among genes. A plausible explanation for the reported results is that ribosomes tend to protect the mRNA molecules from the exosome complexes degrading them; however, additional non-mutually exclusive possible explanations for the reported relation and experiments for their verifications are discussed in the paper.

## Introduction

Regulation of gene expression and protein levels is the result of a series of complex intra-cellular mechanisms which involve interactions with various macromolecules.

The first major step occurs in the nucleus and includes the transcription of the DNA to messenger RNA (mRNA) by RNA polymerase (RNAP), whilst the second major step is the translation of mRNA to protein by the ribosome in the cytoplasm [1]–[3]. Additional regulatory steps include the degradation of the mRNA and protein molecules during the transcription and translation steps [1].

The current study is related to the interaction between two of the gene expression stages: the translation of mRNA molecules to proteins and the degradation of mRNA molecules. In both stages intra-cellular macromolecules interact with mRNA molecules: in the case of translation, mRNA molecules are scanned by ribosomes, the elongation step includes the translation of the mRNA nucleotides (nts) triplets to amino acids [1], [4], [5]; the degradation step includes the digestion of the mRNA molecules by intracellular enzymes [1], [6].

Transcript degradation regulation is not fully understood [7]–[9] and not much is known regarding the enzymes that degrade mRNAs and how they are regulated [7], [10], [11]. mRNA decay can be divided to two broad classes: the first class includes mechanisms of quality control and elimination of potentially toxic proteins; the second class includes mechanisms for shortening half-lives of mRNAs to regulate protein levels of functional protein [7]. The current study is mainly related to the second class.

The nonsense-mediated mRNA decay (NMD) is an example of a mechanism related to the first class; the regulation of NMD appears to be restricted to newly synthesized transcripts unlike most mRNA decay pathways. NMD, which occurs in all the studied eukaryotes [7], eliminates mRNAs that prematurely terminate translation, examples of those degradation pathways are degradation of faulty mRNAs associated with stalled ribosomes and degradation of mRNAs lacking a stop codon or having a premature one [7], [9], [12]–[14].

Eukaryotic mRNAs half-lives vary from several minutes to over 24 hours [2]; changes in mRNA stability are eventually reflected in protein abundance, hence the stability of mRNAs is a substantial phase in the regulation of gene expression and protein abundance in all organisms [2], [15].

The degradation of mRNAs can be modulated in response to various developmental, environmental and metabolic signals [1], [6], [7], [15], and was found to be related at least partially to various sequence elements throughout the mRNA [2], [7], [8], [10]. These include common features of most transcripts: the cap structure at the 5′ end and the poly (A) tail at the 3′ end in eukaryotes, which function to prevent mRNA degradation and improve translation efficiency[15], as well as specific sequences, which can be found at different locations in the mRNA [2], [10], [16], [17]. In eukaryotes, such as *Saccharomyces cerevisiae*, intrinsic mRNA decay initiate with deadenylation that causes the shortening of the poly(A) tail at the 3′ end of the mRNA, followed by the removal of the cap at the 5′ end by the decapping enzyme, which leads to a rapid 5′→3′ degradation of the mRNA by an exoribonuclease [18]. Deadenylation-dependent decapping is thought to be the most common decay pathway for degrading wild-type *S. cerevisiae* mRNAs [18]. The mRNA degradation mechanism in prokaryotes such as *Escherichia coli* is different: Cells contain multiple endoribonucleases and 3′ exoribonucleases, but no 5′ exoribonucleases [19]–[21]. Because these 3′ exonucleases are prevented from being activated by the stem-loop structures that typically are present at the 3′ ends of *E. coli* mRNAs [19], [22], [23], it seems that endonucleolytic cleavage generally precedes 3′ exonucleolytic attack [19]. The endonuclease most important for mRNA decay in *E. coli* is ribonuclease (RNase) E, an essential enzyme that cleaves RNA within single-stranded regions that are AU-rich [19], [24]–[26]. In *E. coli*, RNA degradation often begins with conversion of the 5′-terminal triphosphate to a monophosphate, creating a better substrate for internal cleavage by RNase E [19]. In *Bacillus subtilis* and other bacteria lacking RNase E, 5′ end-dependent RNA degradation is triggered by enzyme that converts the 5′-terminal triphosphate of RNA to a monophosphate [19], resulting in an intermediate that is degraded by the 5′ exonuclease activity of RNase J [19].

Several previous systems biology studies aimed at modeling and understanding the way mRNA degradation is encoded in transcripts; for example, Shalgi et al., 2005 [8] presented a catalog of *53* sequence motifs in the 3′ UTR of *S. cerevisiae* that are associated with either increased or decreased transcript stability.

The degradation rate of mRNA molecules is also determined by specific signaling proteins in response to physiological stimuli [2], [7]. Thus, mRNA stabilization may require appropriate mRNA folding, as it is necessary for signal-induced stabilization, or interactions between different trans-acting factors that modulate mRNA decay [2].

Previous studies revealed the gene expression regulation of various functional gene groups via mRNA degradation: it was shown that genes related to transcription, cell cycle, mRNA processing, apoptosis, signal transduction and development tend to have short mRNA half-lives, whereas genes involved in carbon and nitrogen metabolism, protein biosynthesis, extracellular matrix, cytoskeleton and housekeeping enzymes tend to have stable mRNAs with long half-lives [9], [27].

In the current study we aimed at understanding if higher ribosomal density contributes to higher mRNA half-life of transcripts in *S. cerevisiae* and to quantify this relation.

A previous study [26] reviewed the influence of ribosome binding and translation on the bacterial mRNA decay. Their findings suggest that association of ribosomes tends to protect mRNA from ribonuclease attack [26]; specifically, they suggest that closer spacing of translating ribosomes on bacterial mRNA can mask internal cleavage sites within the coding and non-coding regions of the mRNA, protecting the mRNA molecule from cleavage.

In addition to the fact that ribosome-free or “naked” bacterial mRNA is known to be efficiently degraded, a later study [28] found a clear correlation between the functional mRNA half-life in bacterial *lacZ* gene [28] and the ribosome spacing in the mRNA region approximately between codon *20* and codon *45* [28]. This segment often represents the two ribosomes closest to the 5′ end of the mRNA at any time.

Here we generalize the initial studies in the field in four major directions: first, we analyze, for the first time, the relation between ribosomal density and mRNA degradation in a eukaryote (*S. cerevisiae*). There are various differences between bacteria and eukaryotes transcription and degradation, such as the fact that there is no physical separation between the transcription and the translation steps in bacteria but there is a separation in eukaryotes; in addition, as mentioned above the mRNA degradation pathway in bacteria and eukaryotes is different; thus, it is not trivial that the same relation occurs both in bacteria and eukaryotes.

Second, we perform *large scale* analyses aiming at understanding this relation based on thousands of *endogenous S. cerevisiae* genes; third, our comprehensive study includes refined questions such as understanding the effect of ribosomes local density, the expression levels of the genes, and gene functionality on mRNA degradation rate; finally, we supply a *quantitative* estimations related to the effect of ribosomal density on mRNA half-lives.

## Results

**Figure 1:**
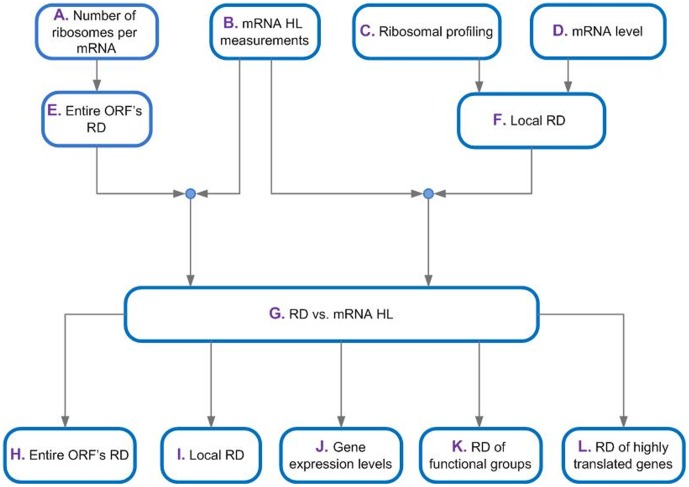
The flow chart and summary of the study. **A.–D.** The analyses are based on large scale measurements of four major variables: The number of ribosomes per mRNA, ribosomal profiling, mRNA levels, and mRNA half-life. These data were used to obtain ribosomal density along the entire ORF (**E.**) and at a resolution of single nucleotide (**F.**) respectively, and was compared to mRNA half-life (**G.–L.**). **H.–L.** We studied the relation between ribosomal density and mRNA half-life (**G.**) at five levels of RD resolution: the RD of the entire ORF (**H.**), the local RD in different parts of the ORF (**I.**), the RD when the gene expression levels are controlled (**J.**), the RD of functional gene groups (**K.**), and the RD of highly translated genes (**L.**).

The current study includes the following stages: first we show that mean ribosomal density (RD) has positive correlation with mRNA half-life (HL); at the next step, we study the ribosomal density at a single nucleotide resolution, showing that the ribosomal density across the ORF contributes to the effect on mRNA half-life; at the next step, we show that the relation between mRNA half-life and ribosomal density cannot be explained only by gene expression levels (i.e. independent selection of higher half-life and ribosomal density in genes with higher gene expression); then, we show that the reported relation between mRNA half-life and ribosomal density is not function specific as it occurs in many functional gene groups in *S. cerevisiae*; Finally, we show that the positive relation between ribosomal density and stability remain significant also for transcripts that undergo many translation events (see Fig. 1); as we will explain later, this result support the conjecture that ribosomal density affect mRNA half-life and not vise-versa.

The analyses in this study were focused on a set of *1,525* highly translated genes with reliable measurements of ribosomal densities at a single nucleotide resolution (that were also analyzed in previous studies in the field; see Materials and Methods and [29]) for two main reasons: First, in the case of lowly translated genes (which usually also have low mRNA levels) the reliability of the measurements and thus the signal to noise is relatively low; second, in the case of genes with very low number of ribosomes we expect that other factors other than ribosomal density will dominate the effect on mRNA half-life, significantly blurring the contribution of ribosomal density. Indeed, we did not find the strong relation that is reported here, when we analyzed lowly translated genes (data not shown).

The RD data were compared to mRNA HL data, which were averaged from five different experiments to filter noise and experimental specific biases of mRNA HL measurements (details in the Materials and Methods section).

### Positive correlation between ribosomal density and mRNA stability in *S. cerevisiae*

We start with the coarsest question: is there any correlation between the number of ribosomes on the mRNA divided by its length (i.e. ribosomal density) and mRNA half-life? In order to answer this question, we tested whether there is a correlation between the genes' RD [30] and mRNA HL ([6], [31]; see Materials and Methods).

**Figure 2:**
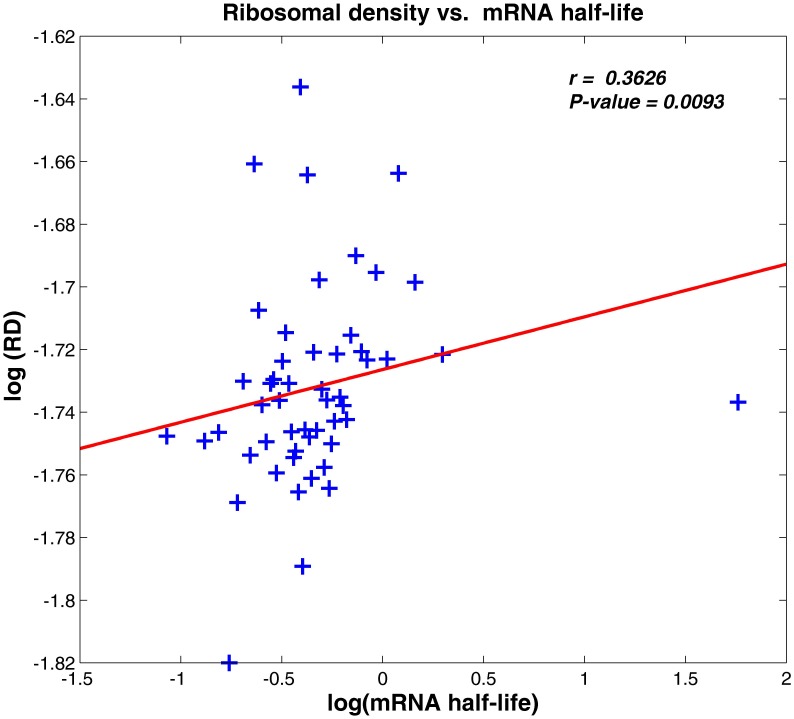
Ribosomal density versus mRNA half-life. Both axes are in logarithmic scale (*r = 0.3626, P-value  = 0.0093*; data were binned to *51* bins to filter noise; details in Materials and Methods). The red line indicates the inferred linear relation between the ribosomal density and mRNA half-life.

The resultant correlation appears in Figure 2. As can be seen, there is indeed a significant positive relation between the ribosomal density of a transcript and its stability (*r = 0.3626*, *P-value  = 0.0093; 51* bins).

When dividing the genes to top (first group) and bottom (second group) *20%* according to the total ORF's RD (the median RD of the first group is *67%* higher than the median RD of the second group; data were taken from [30], more details in the Method section), the half-life median of the genes from the top *20%* group is ∼*10%* higher than the half-life median of the genes from the bottom *20%* group (*P-value  = 0.0228*); data were taken from [31] and [6]. Similar results were obtained based on the ribosomal profiling data at a single nucleotide resolution from ([29], [32]; see Materials and Method): the genes were similarly divided according to the top and bottom *20%* total ORF's RD average (the median RD of the first group is *78%* higher than the median RD of the second group), the half-life median of the genes from the top *20%* is ∼*25%* higher than the half-life median of the genes from the bottom *20%* (*P-value  = 2.6191·10^−10^*; see also Figure S9). This result indicates that genes with higher ribosomal density tend to have longer half-life.

### Ribosomal Density at Single Nucleotide Resolution positively correlates with mRNA Half-life

**Figure 3:**
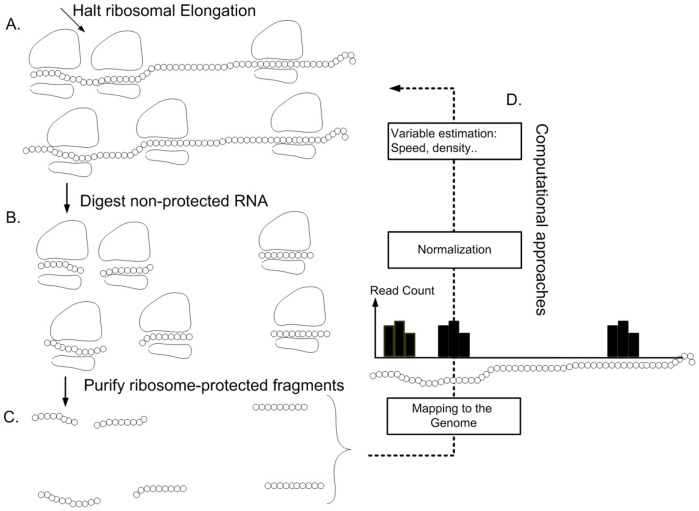
Description of the ribosomal profiling approach : **A**. Cells are treated with cycloheximide (for example) to arrest translating ribosomes; **B**. RNA fragments that are protected from RNases by the ribosomes are isolated and **C**. processed for Illumina high-throughput sequencing. **D**. The next steps are computational – reads are mapped to the ORFs of the analyzed organism. Ribosomal footprint reads of a certain codon are generated when the codon is covered by ribosomes. Thus, highly translated genes tend to create a higher number of reads.

The most promising approach for studying gene translation is the novel ribosomal profiling method [29]. It provides a quantitative measure of the translation status of each nucleotide in the genome at any given moment (see Fig. 3). Cells are treated with cycloheximide (for example) to arrest translating ribosomes; RNA fragments that are protected from RNases by the Ribosomes, are isolated and processed for Illumina high-throughput sequencing. The next steps are computational and include mapping to the ORFs of the analyzed organism. Ribosomal footprint reads of a certain codon are generated when the codon is covered by ribosomes. Thus, highly translated genes tend to create a higher number of reads.

In order to better understand the relation between local ribosomal density and mRNA stability, we generated a RD profile for the *1,525* genes using ribosomal profiling data from [29], [32], which were divided by the yeast mRNA levels (averaged from [29] and [32]), in order to obtain the RD per mRNA (see details in Materials and Method section). The final data include for each gene the ribosomal density along the transcript at single nucleotide resolution.

**Figure 4:**
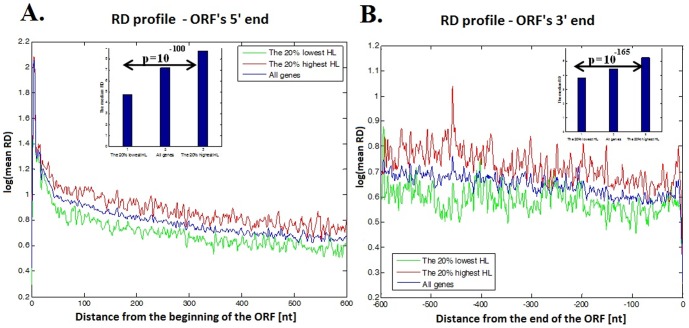
Ribosomal density profile for highly translated genes at single nucleotide resolution. **A**. The first *600* nts; all genes are aligned to the ORF's 5′ end. **B**. The last *600* nts; all genes are aligned to the ORF's 3′ end. The y-axes represent the mean RD in logarithmic scale at specific locations along the ORF; the x-axes represent the location of a nucleotide measured as the distance from the ORF's 5′ end (positive numbers at **A**.) or distance from the ORF's 3′ end (negative number at **B**.). The red line describes the *20%* of genes with the longest mRNA half-life; the green line describes the *20%* of genes with the shortest mRNA half-life, and the blue line describes all the genes. The inset in each plot includes the RD median for each group of genes, from left to right: the *20%* of the genes with the shortest half-life, all genes, and the *20%* of the genes with the longest half-life. The number above the arrow is the P-value corresponding to the Wilcoxon rank sum test between the local averaged RD of genes with the *20%* longest and shortest half-life.

Figure 4 includes the mean ribosomal density profiles for genes with top/bottom *20%* mRNA HL (see Materials and Method).

As can be seen in Figure 4, in every location of the first *600* nucleotides in the ORF's 5′ end, and of the last *600* nucleotides in the ORF's 3′ end, the local averaged RD of the group including the *20%* genes with the longest mRNA half-life (red line in Fig. 4) tend to have higher local ribosomal density than the group of the *20%* genes with the shortest mRNA half-life (green line in Fig. 4, see also zoom in plots in Fig. S3). Specifically, the top *20%* of the genes with the longest mRNA half-lives have a median half-life that is ∼*84%* higher than the median of the *20%* of the genes with the shortest mRNA half-lives. Moreover, the medians of the ribosomal footprint read counts per mRNA for the *20%* genes with the top half-life at the ORF's 5′ and 3′ end are ∼*33%* and *28%* (respectively) larger than those of the *20%* of the genes with the bottom half-life.

In addition, we calculated the P-value based on the Wilcoxon rank sum test between the local averaged RD of genes with *20%* top and bottom mRNA half-life in the ORF's 5′ and 3′ end (see the insets of each plot in Fig. 4). In both ends of the ORF the RD median of the genes group with the longest mRNA half-life is significantly higher than the RD median of the genes group with the shortest half-life (*P-value  = 10^−100^ and P-value  = 10^−165^* for the ORF's 5′ and 3′ end respectively).

**Figure 5:**
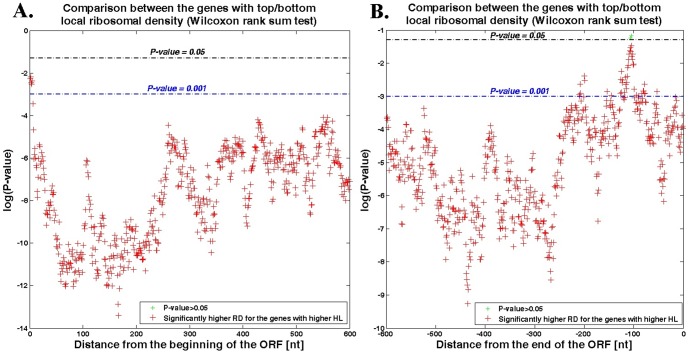
Wilcoxon rank sum test between mRNA half-lives of genes with top and bottom *20%* RD for each *40* nts sliding window. **A**. The ORF's first *600* nts; genes are aligned to the ORF's 5′ end. **B**. The ORF's last *600* nts; genes are aligned to the ORF's 3′ end. The x-axes represent the location of the sliding window downstream to the aligned ORF's 5′ end (positive numbers at **A**.) and upstream to the aligned ORF's 3′ end (negative number at **B**.) respectively; the y-axes represent the log (Wilcoxon test P-value); the black line indicates that *P-value  = 0.05*; the blue line indicates that *P-value  = 0.001*. The green cross indicates that there is no significant difference between the half-life medians of the two groups; the red cross indicates that the half-life median of the genes group with higher RD is significantly higher than the one of the genes group with lower RD (*P-value ≤0.05)*. There were no positions with significant signal in the opposite direction (i.e. genes with higher RD that have significantly lower HL).

Next, we aimed at answering the counter question: do the genes with higher local ribosomal density (in different parts of the transcript) tend to have longer half-life? Similarly to the previous analysis, we first aligned all the genes to their ORF's 5′ and 3′end, and calculated for each gene, using the ribosomal profiling data from ([29], [32]; see Materials and Method), the mean RD of *40* nts sliding windows (slide of single nt). For each window we divided the genes to two groups with *20%* highest and lowest RD, and performed a Wilcoxon rank sum test to support the hypothesis that there is a significant difference between the mRNA HL median of these two groups; data were taken from ([6], [31]; see Materials and Method). The results appear in Figure 5.

As can be seen in Figure 5, in every location of the sliding window in the ORF's 5′ end along the first *600* nucleotides, the half-life median of the genes from the top *20%* RD is higher than the one of the genes from the bottom *20%* RD. The same result is obtained in the ORF's 3′ end (see also Figure S4).

**Figure 6:**
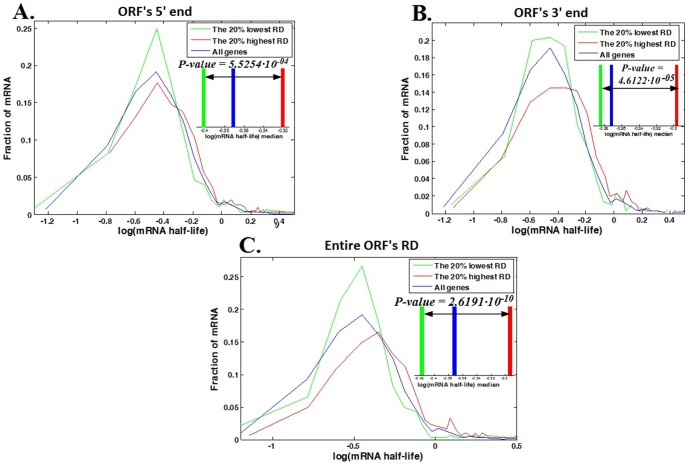
mRNA half-life distributions. Half-life distributions of the genes from the bottom *20%* RD (green curve), top *20%* RD (red curve), and of all genes (blue curve). The inset in each plot includes the median of each curve, which is represented by the intersection with the x-axis of a vertical line with the appropriate color: green, red and blue lines that indicate the half-life medians of the genes from the bottom and top *20%* RD and of all genes respectively; for a better visualization, the graphs are based on the log (mRNA HL) values. The number above the arrow in each inset is the P-value corresponding to the Wilcoxon rank sum test between the mRNA HL of genes with the top and bottom *20%* RD. **A**. Half-life distributions at the ORF's 5′ end according to the genes RD average of the first *50* nucleotides downstream the ORF's 5′ end. **B**. Half-life distributions at the ORF's 3′ end according to the genes RD average of the last *50* nucleotides upstream the ORF's 3′ end. **C**. Half-life distributions according to the genes total average of the ORF's RD.

The distributions of the mRNA half-life values for genes from the top and bottom *20%* RD in the ORF's 5′ end, ORF's 3′ end, and the entire ORF are shown in Figure 6 (details about the calculation of the ORF's ends RD can be found in the Materials and Methods section).

From Figure 6, which represents the half-life distributions according to the RD at the ORF's 5′ and 3′ ends and along the entire ORF, it can be seen that the half- life distribution of the genes from the top *20%* RD (red curve in Fig. 6A–C) is shifted to the right (higher) relative to the distribution of the genes from the bottom *20%* RD (green curve in Fig. 6A–C), resulting in a higher half–life median of the genes with higher RD: the *20%* of genes with the highest averaged RD have a median RD that is ∼*89%, 87%* and *78%* higher than the median of the *20%* of genes with the lowest averaged RD at the ORF's 5′ end, 3′ end and the entire ORF respectively; the half-life medians (red vertical line in the insets of Fig. 6A–C) of the genes from the top *20%* RD are ∼*17%* (*P-value  = 5.5254·10^−04^*), *18%* (*P-value  = 4.6122·10^−05^*) and *25%* (*P-value  = 2.6191·10^−10^*) higher than the medians of the genes from the bottom *20%* RD (green vertical line in the insets of Fig. 6A–C) in the ORF's 5′ end, 3′ end and the entire ORF respectively.

### The Relation between mRNA half-life and ribosomal density cannot be explained by gene expression levels

It is possible that positive correlation between mRNA half-life and ribosomal density is due to the fact that highly expressed genes are independently selected for high ribosomal density and high mRNA stability. In order to refute this possibility we computed partial correlations between the ribosomal density of a transcript and mRNA half-life given the protein abundance (PA) of the genes. The ribosomal density of a transcript was calculated in a various ways: 1) using the number of ribosomes per transcript according to [30]; using the ribosomal profiling data from [29], [32] that were divided by the yeast mRNA levels (averaged from [29] and [32]) in order to obtain the RD per mRNA, which for each gene the RD were averaged over 2) the entire ORF, 3) the ORF's 5′ and 4) 3′ ends (see Materials and Methods section).

**Figure 7:**
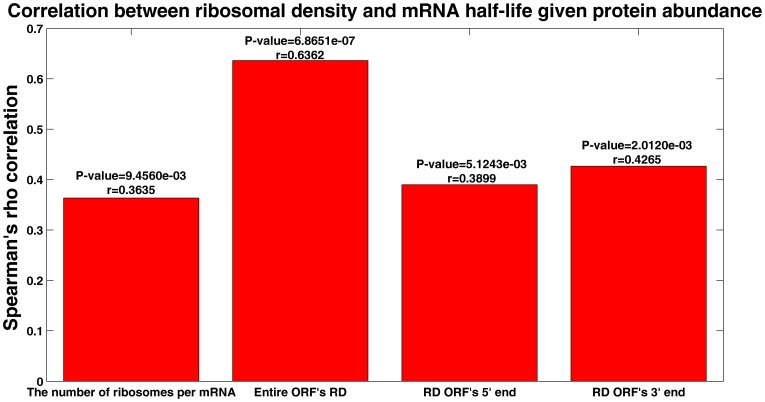
Ribosomal density versus mRNA half-life given the protein abundance (binned data; details in Materials and Methods). The RD were calculated as: 1) the number of ribosomes on the mRNA [30] divided by its length, 2) averaging the ribosomal profiling data from [29], [32] over the entire ORF, 3) the ORF's 5′ end –the first 50 nt and 4) the ORF's 3′ end – the last 50 nt.

The resultant correlations appear in Figure 7. As can be seen, the significant positive relation between the ribosomal density of a transcript and its stability is still maintained even when the protein abundance is controlled. This result obtained for both the number of ribosomes per mRNA data [30] and the ribosomal profiling data [29], [32] at different locations along the ORF and support the conjecture that the positive relation between mRNA half-life and ribosomal density can't be fully explained via an independent evolutionary selection which is related to the genes protein abundance.

### The positive relation between ribosomal density and stability is not function specific

In order to show that the positive relation between RD and mRNA HL, which was described above, is global and not related to the regulation of some specific functional gene groups we used Gene ontology (see also the list of the functional gene groups for each ontology at Table S5) and performed the aforementioned analyses for different GO groups separately (see Materials and Methods).

**Figure 8:**
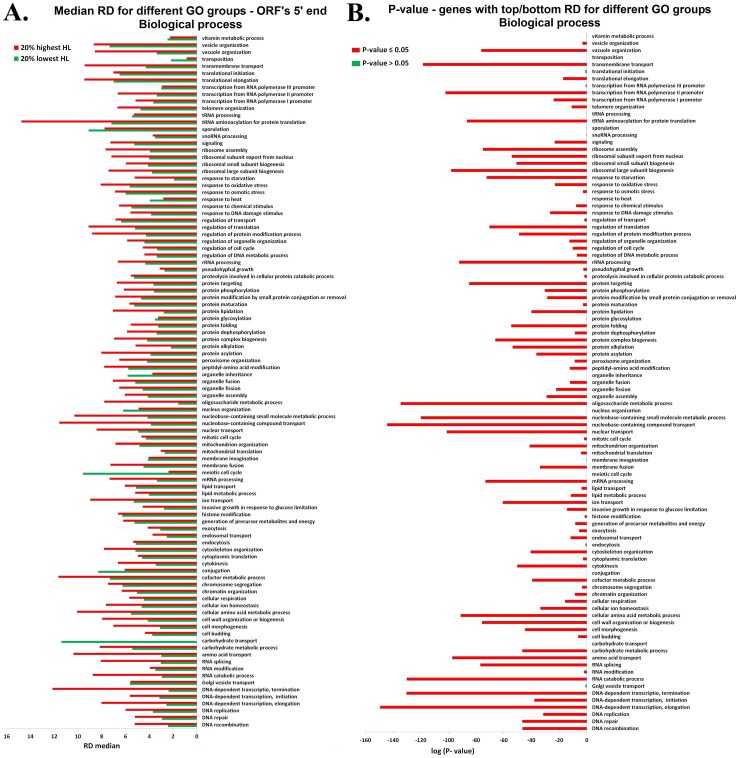
Biological process GO: RD profile at single nucleotide resolution, of the first *600* nts when all genes are aligned to the ORF's 5′ end : **A**. The RD median of the ORF's 5′ end RD profiles: the red/green bars represent the RD median of the *20%* of the genes with top/bottom half-life. **B**. Wilcoxon rank sum test between the RD profiles of the genes from the top and bottom *20%* half-life for different functional genes groups. The x-axis represents the log (Wilcoxon test P-value) and the y-axis represents the functional genes group (see Table S5). Red bars indicate that *P-value ≤0.05* whereas green bars indicate that *P-value >0.05*.

**Figure 9:**
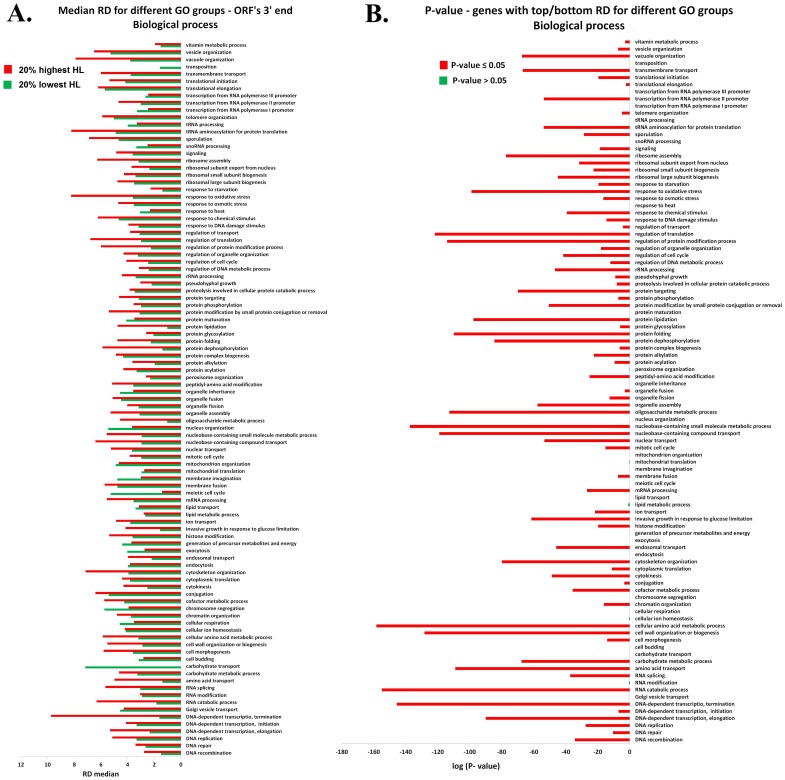
Biological process GO: RD profile at single nucleotide resolution, of the last *600* nts when all genes are aligned to the ORFs 3′ end : **A**. The RD median of the ORF's 3′ end RD profiles: the red/green bars represent the RD median of the *20%* of the genes with top/bottom half-life. B. Wilcoxon rank sum test between the ribosomal densities profiles of the genes from the top and bottom *20%* half-life for different functional genes groups. The x-axes represent the log (Wilcoxon test P-value) and the y-axes represent the functional genes group (see Table S5). Red bars indicate the *P-value ≤0.05* whereas green bars indicate the *P-value >0.05*.

As can be seen in Figures 8–9 and Figures S5–S8, our analyses demonstrate that indeed the reported phenomenon is not function specific.

As performed in the previous sections for the entire set of genes, we divided each functional group (the functional groups in Figures 8–9 and Figures S5–S8 are in the same order as in Table S5 for the readers' convenience) to two sets of genes with *20%* top and bottom half-life. For each of the top/bottom sets of the functional groups, we computed the median RD over the ribosomal profiling data and performed Wilcoxon rank sum test between the RD profiles of the two sets (details in the Materials and Methods section). Figures 8–9 represent the biological process ontology analyses; as can be seen; both in the ORF's 5′ (Fig. 8) and 3′ end (Fig. 9) the RD median of genes with the longest half-life tends to be significantly higher than the RD median of genes with the shortest half-life in the majority of the functional groups. Specifically, in *82* and *73* out of the *99* biological process groups in the case of the 5′ and 3′ end of the ORFs respectively. The corresponding Wilcoxon P-values were significant (details, including a list of GO terms which did not pass the Wilcoxon test, in Table S4). Similar results were obtained for the two other gene ontology domains: Molecular function and Cellular component (see Fig. S5–S8 and Table S4).

### The positive relation between ribosomal density and stability remain significant also for transcripts that undergo many translation events

It is also possible that a relatively large pool of nuclear mRNA, which is physically separated from the translational apparatus, exists. This nuclear pool consists mostly of newly-made RNA being processed and exported. An mRNA that is turned over quickly in the cytosol will spend more of its life as a nuclear RNA and thus at steady state less of this RNA will be accessible to ribosomes, leading to a negative correlation between ribosomal density and mRNA turnover. The aim of this subsection is to evaluate the plausibility of this explanation.

First, though we did not find estimation of the relative number of mRNA molecules in the nuclear we believe that it is at least 1–2 orders of magnitude lower than the mRNA levels outside of it. For example, the size of the nuclear was estimated to be only ∼7 % out of the cell volume (see, for example [33]). Thus, we believe that the effect of intra-nuclear mRNA levels on the measurements and analyses performed here is negligible.

Second, we estimated the relation between ribosomal density and mRNA half-life in transcripts that undergo many translation events, showing that it is still significantly positive. These genes are expected to be in steady states (or very close to steady) in terms of the number of ribosomes translating them at a time point; thus, increasing their half-life should not affect their ribosomal density and the explanation above is less plausible.

To this end we analyzed the relation between mRNA half-life and ribosomal density in two groups of genes. The first group includes the genes that their ratio between the number of proteins and mRNA levels is larger than *100* (details in the Materials and Methods section); the Spearman correlation between the binned data of mRNA HL and the entire ORF's RD, in this group is *r  = 0.5493 (P-value  = 4.8367·10^−05^, 50* bins; the correlation for non binned data is *r  = 0.1164*, *P-value  = 1.0489·10^−04^*). The Wilcoxon rank sum test between mRNA half-life values for genes in this group from the top and bottom *20%* of the entire ORF's RD was calculated (details in the Materials and Methods section): the *20%* of genes with the highest averaged entire ORF's RD have a median RD that is ∼*78%* higher than the median of the *20%* of genes with the lowest RD; the half-life median of the genes from the top *20%* RD is ∼*25%* (*P-value  = 3.4282·10^−05^*; see also Figure S10) higher than the median of the genes from the bottom *20%* RD.

The second group includes genes that their ratio between mRNA half-life and the estimated translation time is larger than *50* (details in the Materials and Methods section); the Spearman correlation between the binned data of mRNA HL and the entire ORF's RD in this group is *r  = 0.4696* (*P-value  = 0.0095, 50 bins*; the correlation for non binned data is *r  = 0.1062*, *P-value  = 0.0017*). The Wilcoxon rank sum test between mRNA half-life values for genes in this group from the top and bottom *20%* of the entire ORF's RD was calculated (details in the Materials and Methods section): the *20%* of genes with the highest averaged entire ORF's RD have a median RD that is ∼*79%* higher than the median of the *20%* of genes with the lowest RD; the half-life median of the genes from the top *20%* RD is ∼*17%* (*P-value  = 2.2590·10^−04^*; see also Figure S11) higher than the median of the genes from the bottom *20%* RD.

The analyzing results of the above two groups show that the relation between ribosomal density and mRNA half-life in transcripts that undergo many translation events, is still significantly positive.

## Discussion

In this study we demonstrate for the first time that there is an interaction between two gene expression stages, mRNA decay and gene translation based on whole genome analyses of *S. cerevisiae*.

According to previous small scale studies on bacteria [26], [28], “naked” mRNAs that are free from ribosomes tend to be more degradable than mRNAs covered with ribosomes. However, it is known that the degradation mechanisms in bacteria and eukaryotes are different [2], [10], [11], [14], [17], [19], [26], [34]. Our analyses on *S. cerevisiae* genes, using ribosomal density at single nucleotide resolution, support these previous studies and reveal that also in eukaryotes, such as *S. cerevisiae*, mRNA molecules with higher ribosomal density along their ORF tend to be more stable with longer mRNA half-life. Specifically, based on the large scale data analyzed here, we were able to estimate the effect of higher RD on increasing mRNA HL: an increase of *∼78%* in ribosomal density yields an increase of about *25%* in mRNA half-life in the analyzed organism.

Specifically, we provide various pieces of evidence that support the conjecture that indeed the reported relation between RD and mRNA HL is robust: first, we analyzed several datasets of mRNA HL that were obtained in different experimental conditions to remove biases [6], [31], and several RD measurements based on two technologies [29], [30], [32]; second, we demonstrated that the reported relation is due to RD along the entire ORF and not in specific locations; third, we showed that the positive relation between mRNA HL and its RD is not a consequence of an evolutionary selection of the gene's protein abundance as we controlled the PA when obtaining the correlation between mRNA HL and RD; fourth, we detected the reported relation in most of the gene functions, demonstrating that it is not function specific.

It is impossible to prove causality based on the analyses of endogenous genes reported in this study. Thus, several theoretical (non mutually exclusive) relations are possible: 1) higher RD increase mRNA HL in a direct way (e.g. by protecting the mRNA from exonucleases); 2) genes with higher mRNA HL are selected for higher RD; for example, due to the fact that these are highly expressed genes and both increasing the translation initiation rate (ribosomal density) and increasing mRNA stability should contribute to higher protein levels; 3) It is also possible that a pool of nuclear mRNA physically separated from the translational apparatus exists. This nuclear pool consists mostly of newly-made RNA being processed and exported. An mRNA that is turned over quickly in the cytosol will spend more of its life as a nuclear RNA and thus at steady state less of this RNA will be accessible to ribosomes, leading to the negative correlation between ribosome density and mRNA turnover; We believe that relation 1) is the best explanation (or at least part of the explanation) for the observed positive correlation between RD and mRNA HL in *S. cerevisiae* due to five major reasons: first, we showed that statistically the positive relation between the transcript stability and its ribosomal density cannot be explained by the genes' protein abundance; thus, relation 2) alone cannot explain the observed results; second, our analyses support the hypothesis that the obtained correlation is also significant for genes that undergo many translation events. The ribosomal density profile of these genes is expected to be in (or very close to) steady state (i.e. should not be changed if their half-lives were lengthened), therefore relation 3) is a less plausible explanation for the obtained positive correlation between transcript RD and its stability; third, though degradation mechanism in bacteria and eukaryotes are different, the fact that such a causal relation has been shown based on small scale analyses in *E. coli* [26], [28] supports the results reported here; forth, the fact that the reported results are not function specific support the conjecture that the relation is direct and not via additional (possibly function/pathway specific) variables; and fifth, previous studies have shown that reductions in the rate of translation initiation, which should cause a decrease in the transcript's ribosomal density, frequently lead to accelerated mRNA deadenylation and subsequent decapping, leading to mRNA degradation [34]–[36], whereas inhibition of translation elongation or termination, which frequently causes an increase of the transcript's ribosomal density, generally leads to mRNA stabilization [34], [35], [37], [38]. Though, these studies do not directly discuss the possibility that the reported relations are related to changes in ribosomal densities, this is a plausible explanation for at least part of these results which is implied by the authors of some of these papers.

A possible experiment for validating causality can include generating a synthetic library of a reported gene in the following way: All the variants are identical but their Kozak sequence surrounding the START codon [39], [40] that varies among the different variants; a more optimal Kozak sequence corresponds to a higher initiation rate and thus higher RD, but it is not expected to affect degradation efficiency or the transport of mRNA from the nucleus. The mRNA HL and the RD will be measured for each variant using the technique mentioned in previous studies [6], [29]–[32], [41].

The fact that all variants are almost identical will enable to validate if indeed relation 1) is true as it is clearly control for the other explanations.

The results reported in this study demonstrate that gene expression stages are not independent, and there are cross-talk and overlap between the different stages; previous studies have shown that other stages of gene expression are also strongly connected: for example, it was shown that there is an interaction between RNA polymerases and ribosomes in prokaryotes [42], and that translation initiation and elongation steps are coupled in various ways [43].

One major conclusion from our study is that an increase in ribosomal density due to evolutionary selection or via engineering of synthetic genes will probably have a positive effect also on the half-life of the mRNA molecule.

Finally, when relevant large scale measurements of RD, mRNA HL, and mRNA levels will be amassed, it will be interesting to perform similar analyses in additional organisms (eukaryotes, bacteria, and archaea) and in various conditions to estimate the strength of the reported relation across the tree of life.

## Materials and Methods

### S. cerevisiae sequences

The *S. cerevisiae* genome, including *5,861* genes, was downloaded from the Biomart Ensembl database (http://asia.ensembl.org/). *S. cerevisiae* 5′UTRs and 3′UTRs were obtained from [44].

### mRNA Half-life (HL) datasets

*S. cerevisiae* mRNA half-life data were taken from [31] and [6].

The study of [31] included mRNA decay experiments under two conditions: 1) natural mRNA molecules and 2) mRNAs with shortened Poly (A) tails. The Spearman correlation between these two experiments is 0.6226 (*P-value <10^−323^*). The mRNA half-life histograms of each experiment are shown in Figure S1.

The study of [6] included mRNA decay experiments under two different environmental conditions: exposure to hydrogen peroxide (H_2_O_2_), which induces an oxidative stress, and exposure to methyl methanesulfonate (MMS) which induces DNA damage. A reference decay experiment was also carried out without applying any of the above stresses. The Spearman correlations between the different measurements are presented at Table S1. The histograms of each experiment are plotted in Figure S2.

The Spearman correlation between all the *S. cerevisiae* mRNAs half-life datasets from [31] and [6] are presented in Table S2.

As can be seen from Table S2, all *S. cerevisiae* mRNAs half-life datasets are correlated, hence all of them were averaged together as this average represents the typical yeast decay over various conditions. Specifically, by averaging we filter some of the noise and experimental specific biases.

The number of genes in each dataset is as follows:

1. Natural mRNA – *4,236* genes.
2. mRNAs with shortened Poly (A) – 5,093 genes.
3. Oxidative stress – *5,448* genes.
4. MMS stress – *5,214* genes.
5. Reference – *5,383* genes.

To consider the different mRNA HL mean in each dataset, we averaged the different measurements in the following manner: first, the measurements of each dataset were divided by its mean; at the second step, the different (normalized) datasets were averaged. These are the mRNA HL data that have been used throughout the paper. Nevertheless the reported results are similar also when considering each mRNA HL databases separately (see table S6).

### mRNA levels datasets

mRNA levels data were collected from [31], [29], and [32]. In each dataset the raw data were given as the density of mRNA fragments generated by deep sequencing per nucleotide for each gene. The mRNA level for a gene was calculated as the mean of its mRNA density. In the next step, as in the case of the mRNA HL measurements, each dataset was normalized by its mean, and then the normalized datasets were averaged. These are the mRNA levels used throughout the paper.

The spearman correlations between the different datasets are shown in Table S3 (all correlations are higher than *0.58* (*P-value <10^−323^*)).

The number of genes in each dataset is as follows:

1. Wang et al., 2002–*5,126* genes.
2. Ingolia et al., 2009–*5,295* genes.
3. Brar et al., 2012–*5,860* genes.

### Protein abundance (PA) datasets

Large-scale data of PA in a YPD condition from the study by Ghaemmaghami et al., 2003 [45] were considered (a total of *3,839* genes). This is the protein abundance used throughout the paper.

### Ribosomal densities (RD)

#### Ribosomal density at a resolution of entire genes

These data were taken from [30]. These data originally included measurements of the number of ribosomes per mRNA, and we divided it by the length of each gene in order to obtain ribosomal densities. This dataset includes *5,181* genes. From these data a set of *1,525* highly translated genes with reliable measurements, were taken for analyses (see the next paragraph and [29]).

#### Ribosomal density at a resolution of single nucleotide

Ribosomal profiling data were taken from [32] and [29]. In the case of the data from [29] any gene with fewer than *64* total reads in the first *151* codons was excluded, resulting in *1,525* highly translated genes (the data filtering was performed in the original paper of [29]).

We define the ribosomal position at a codon resolution to be the three nucleotides in the ribosome A-site; thus, we summed the read-counts of the three nucleotides corresponding to the position of the A site of the ribosome.

When averaging the two datasets, only the *1,525* genes common to both datasets were used. In addition, [29] ribosomal profiling data were scaled to [32] ribosomal profiling data by multiplying the read count of each nucleotide by the ratio between the genes read counts total sum of [32] and [29]. At the next step, the RD per nucleotide was averaged across the two datasets. These ribosomal profiling data are proportional to the ribosomal density but also to the mRNA levels of the corresponding genes. Thus, afterwards the averaged ribosomal profiling data were normalized with the mean normalized *S. cerevisiae* mRNA levels, which were averaged from [29] and [32], in order to obtain a measure of ribosomal density for each transcript. These are the ribosomal densities at a single nucleotide resolution that have been used throughout the paper.

### The lists of genes used in different parts of the study

The lists of genes used in different parts of the study appear in table S6.

### Correlation between the ribosomal density of mRNA and mRNA half-life

We perform a Spearman Correlation between the number of ribosomes on the mRNA [30] divided by its length and mRNA Half-life (based on [31] and [6] half-life datasets, as was explained above), in order to assess whether there is a monotonic relationship between these two variables (without any assumption of linearity). The data were binned according to the mRNA half-life with a bin size equals to *30* genes; including in total *51* bins.

### The percent calculation formula

Throughout the paper, the percent calculation of the form “x is %z from y” was done using this formula: 
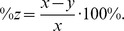


### The mean ribosomal density profiles calculation

First, the genes were divided to three groups: 1) all genes; 2) genes with top *20%* mRNA HL; 3) genes with bottom *20%* mRNA HL [6], [31]. Next, using the ribosomal profiling data from [29], [32] which were divided by the yeast mRNA levels (averaged from [29] and [32]) for each transcript; all ribosomal profiles were aligned to their ORF's 5′ and 3′end. The mean RD for a specific nucleotide was calculated along all genes from the three groups, over the first *600* nts downstream to the aligned ORF's 5′ end (for the 5′ end alignment) and the last *600* nts upstream to the aligned ORF′s 3′ end (for the 3′ end alignment), resulting in the RD profiles for the three groups at two ORF's ends.

### Comparing the mRNA HL medians of genes with high and low RD via a Wilcoxon rank sum test

Using the ribosomal profiling data from [29], [32] which were divided by the yeast mRNA levels (averaged from [29] and [32]) for each transcript; all genes were aligned to their ORF's 5′ and 3′end, in order to calculate for each gene the mean RD of *40* nts sliding windows (slide of single nt). For each window the genes were divided to two groups with *20%* highest and lowest RD, and a Wilcoxon rank sum non-parametric test was performed to support the hypothesis that there is a significant difference between the mRNA HL [6], [31] medians of these two groups. It is important to mention that the reported results remain significant and similar also when we use different cut-offs (*e.g.* 5%, 10%, 20% 25%, 30% instead of 20%; see table S6).

### Calculation of the ORF's ends RD

The RD of the ORF's 5′ and 3′ end were calculated for each gene by averaging the ribosomal profiling data from [29], [32] which were divided by the yeast mRNA levels (averaged from [29] and [32]), over the first/last *50* nucleotides in the ORF's 5′/3′ end.

### Correlation between the ribosomal density of mRNA and mRNA half-life given protein abundance

We perform partial Spearman Correlations between mRNA Half-life (based on [31] and [6] half-life datasets, as was explained above) and the different RD datasets including RD at different locations on the ORF, given the PA (based on [45] PA dataset, as was explained above), when the RD were calculated as: 1) the number of ribosomes on the mRNA [30] divided by its length 2) the entire ORF's RD, 3) RD of the ORF's 5′ end and 4) 3′ end. The last three RD were calculated for each gene by averaging the ribosomal profiling data from [29], [32] which were divided by the yeast mRNA levels (averaged from [29] and [32]), over the entire ORF and the first/last *50* nucleotides in the ORF's 5′/3′ end respectively. The partial Spearman Correlations were done in order to assess whether the monotonic relationship between mRNA HL and RD (without any assumption of linearity) is not a consequence of the PA, meaning that the correlation between these two variables is done when the PA is controlled. For each correlation the data were binned according to the mRNA half-life with a bin size equals to *30* genes; including in total *51* bins.

Similar results were obtained without binning: *r = 0.054* (*P-value  = 0.042*), *r = 0.14* (*P-value  = 1.16·10 ^−7^*), *r = 0.088* (*P-value  = 7.1·10^−4^*), *r = 0.085* (*P-value  = 0.001*); for the cases 1), 2), 3), and 4) mentioned above.

### Analyzing the RD of the functional groups

We used Gene ontology (GO; http://www.yeastgenome.org/cgi-bin/GO/goSlimMapper.pl) to derive all the functional gene groups related to the three ontology domains: Cellular component, Molecular function and Biological process. We divided each functional group (the number of genes in each group appears in Table S5) to two sets of genes with top and bottom *20%* mRNA half-life. For each set and for the total functional group we derived the RD profiles for the first *600* nucleotides when all genes are aligned to the ORF's 5′ end and the last *600* nucleotides when all genes are aligned to the ORF's 3′ end (as described above; see also Fig. 4). Then we calculated the RD median for each set as can be seen in Figures 8A and Figures S5A, S7A for the ORF's 5′ end and Figures 9A and Figures S6A, S8A for the ORF's 3′ end. We performed a Wilcoxon rank sum test between the average RD (at single nucleotide resolution) of genes from the top and bottom *20%* mRNA half-life (the RD profiles), when all genes are aligned to the ORF's 5′end (see Fig. 8B and Fig. S5B, S7B) and 3′end (see Fig. 9B and Fig. S6B, S8B).

### The positive relation between ribosomal density and stability remain significant also for transcripts that undergo many translation events

We considered the genes into two gene groups: the first group includes the genes that their ratio between the number of proteins per mRNA levels (PA/mRNA) is larger than *100*. The mRNA levels used for estimating PA/mRNA is from [29]; the data were normalized such that there will be *60,000* mRNAs in the cell, which is the estimated number of mRNAs in a cell [46]. The number of proteins is the PA that was taken from [45] as was explained above. The total number of genes out of *1,525* in this group is *1,123* genes.

The second group includes genes that their ratio of mRNA half-life and estimated translation time is larger than *50*. The estimated translation rate is *10* codons per second [47]; based on this estimation we estimated the translation elongation time of each ORF based on its length. The mRNA half-life times were based on the YPD condition measurements that were taken from [6] (it is the reference experiment, as was explained above). The total number of genes out of *1,525* in this group is *875* genes.

For these two groups, Spearman correlation was obtained between the mRNA HL measurements in YPD condition [6] and the entire ORF's RD, which was calculated for each gene by averaging the entire ORF's ribosomal profiling data from [29], [32] that were divided by the yeast mRNA levels (averaged from [29] and [32]). The data were binned according to the mRNA half-life with a bin size equals to *30* genes; including in total *50* bins. Moreover, for each of these two groups a Wilcoxon rank sum test was obtained between mRNA half-life values for genes from the top and bottom *20%* of the entire ORF's RD.

## Supporting Information

Figure S1 **Histograms of the two half-life decay experiments from ref.** [31] **study:** (**A**) for natural mRNA molecules and (**B**) for mRNAs with shortened Poly (A) tails.
(JPG)

Figure S2 **Histograms of the different half-life decay experiments data from ref.** [6] **study** – (**A**) a reference experiment and two different environmental conditions: (**B**) exposure to oxidative stress and (**C**) exposure to MMS.
(JPG)

Figure S3 **Ribosomal density (RD) profile for highly translated genes at single nucleotide resolution.** (**A**) The first *200* nts; all genes are aligned to the ORF's 5′ end. (**B**) The last *200* nts; all genes are aligned to the ORF's 3′ end. The y-axes represents the mean RD in logarithmic scale at specific location along the ORF; the x-axes represents the location of a nucleotide measured as a distance from the ORF's 5′ end (positive numbers at (**A**)) or distance from the ORF's 3′ end (negative number at (**B**)). The red line represents the *20%* of genes with the longest half-life; the green line represents the *20%* of genes with the shortest half-life and the blue line represents all the genes.
(JPG)

Figure S4 **Wilcoxon rank sum test between mRNA half-lives of genes from the top and bottom** ***20%*** **RD for each** ***40*** **nts sliding window at a resolution of single nt.** The RD data were calculated as the average RD that were normalized with the mRNA levels averaged from refs. [29], [31], [32] data. (**A**) The ORF's first *600* nts; genes are aligned to the ORF's 5′ end. (**B**) The ORF's last *600* nts; genes are aligned to the ORF's 3′ end. The x-axis represents the location of the sliding window downstream to the aligned ORF's 5′ end (positive numbers at (**A**)) and upstream to the aligned ORF's 3′ end (negative number at (**B**)) respectively; the y-axis represents the log (Wilcoxon test P-value); the black line indicates that *P-value  = 0.05*; the blue line indicates that *P-value  = 0.001*. The green cross indicates that there is no significant difference between the half-life medians of the two groups; the red cross indicates that the half-life median of the genes group with higher RD is significantly higher than the one of the genes group with lower RD (*P-value ≤0.05)*. There were no positions with significant signal in the opposite direction (i.e. genes with higher RD that have significantly lower HL).
(JPG)

Figure S5 **Cellular component GO: RD profile at single nucleotide resolution, of the first** ***600*** **nts when all genes are aligned to the ORF's 5′ end**: (**A**) The RD median of the ORF's 5′ end RD profiles: the red/green bars represent the RD median of the *20%* of the genes with top/bottom half-life. (**B**) Wilcoxon rank sum test between the RD profiles of the genes from the top and bottom *20%* half-life for different functional genes groups. The x-axis represents the log (Wilcoxon test P-value) and the y-axis represents the functional genes group (see Table S5). Red bars indicate that *P-value ≤0.05* whereas green bars indicate that *P-value >0.05*.
(JPG)

Figure S6 **Cellular component GO: RD profile at single nucleotide resolution, of the last** ***600*** **nts when all genes are aligned to the ORFs 3′ end:** (**A**) The RD median of the ORF's 3′ end RD profiles: the red/green bars represent the RD median of the *20%* of the genes with top/bottom half-life. (**B**) Wilcoxon rank sum test between the ribosomal densities profiles of the genes from the top and bottom *20%* half-life for different functional genes groups. The x-axis represents the log (Wilcoxon test P-value) and the y-axis represents the functional genes group (see Table S5). Red bars indicate that *P-value ≤0.05* whereas green bars indicate that *P-value >0.05*.
(JPG)

Figure S7 **Molecular function GO: RD profile at single nucleotide resolution, of the first** ***600*** **nts when all genes are aligned to the ORF's 5′ end**: (**A**) The RD median of the ORF's 5′ end RD profiles: the red/green bars represent the RD median of the *20%* of the genes with top/bottom half-life. (**B**) Wilcoxon rank sum test between the RD profiles of the genes from the top and bottom *20%* half-life for different functional genes groups. The x-axis represents the log (Wilcoxon test P-value) and the y-axis represents the functional genes group (see Table S5). Red bars indicate that *P-value ≤0.05* whereas green bars indicate that *P-value >0.05*.
(JPG)

Figure S8 **Molecular function GO: RD profile at single nucleotide resolution, of the last** ***600*** **nts when all genes are aligned to the ORFs 3′ end**: (**A**) The RD median of the ORF's 3′ end RD profiles: the red/green bars represent the RD median of the *20%* of the genes with top/bottom half-life. (**B**) Wilcoxon rank sum test between the ribosomal densities profiles of the genes from the top and bottom *20%* half-life for different functional genes groups. The x-axis represents the log (Wilcoxon test P-value) and the y-axis represents the functional genes group (see Table S5). Red bars indicate that *P-value ≤0.05* whereas green bars indicate that *P-value >0.05*.
(JPG)

Figure S9 **mRNA half-life distributions based on the data of Arava** ***et al*** **.** Half-life distributions of the genes from the bottom *20%* RD (green curve), top *20%* RD (red curve), and of all genes (blue curve). The inset includes the median of each curve, which is represented by the intersection with the x-axis of a vertical line with the appropriate color: green, red and blue lines that indicate the half-life medians of the genes from the bottom and top *20%* RD and of all genes respectively; for a better visualization, the graphs are based on the log (mRNA HL) values. The number above the arrow is the P-value corresponding to the Wilcoxon rank sum test between the mRNA HL of genes with the top and bottom *20%* RD.
(TIF)

Figure S10 **mRNA half-life distributions based genes that their ratio between the number of proteins and mRNA levels is larger than** ***100*** *.* Half-life distributions of the genes from the bottom *20%* RD (green curve), top *20%* RD (red curve), and of all genes in the group (blue curve). The inset includes the median of each curve, which is represented by the intersection with the x-axis of a vertical line with the appropriate color: green, red and blue lines that indicate the half-life medians of the genes from the bottom and top *20%* RD and of all genes in the group respectively; for a better visualization, the graphs are based on the log (mRNA HL) values. The number above the arrow is the P-value corresponding to the Wilcoxon rank sum test between the mRNA HL of genes with the top and bottom *20%* RD.
(TIF)

Figure S11 **mRNA half-life distributions based genes that their ratio between mRNA half-life and the estimated translation time is larger than** ***50.*** Half-life distributions of the genes from the bottom *20%* RD (green curve), top *20%* RD (red curve), and of all genes in the group (blue curve). The inset includes the median of each curve, which is represented by the intersection with the x-axis of a vertical line with the appropriate color: green, red and blue lines that indicate the half-life medians of the genes from the bottom and top *20%* RD and of all genes in the group respectively; for a better visualization, the graphs are based on the log (mRNA HL) values. The number above the arrow is the P-value corresponding to the Wilcoxon rank sum test between the mRNA HL of genes with the top and bottom *20%* RD.
(TIF)

Table S1 **Spearman correlation (and P-values) between the different half-life decay experiments data from ref.** [6] **.** - a reference experiment and two different environmental conditions: exposure to oxidative stress and exposure to MMS.
(PDF)

Table S2 **Spearman correlation (and P-values) between the datasets of the different half-life decay experiments from refs.** [6], [31] **. studies.**
(PDF)

Table S3 **Spearman correlation (and P-values) between the different mRNA levels datasets from refs.** [29], [31], [32] **. studies.**
(PDF)

Table S4 **The functional groups divided to the three GO domains (1^st^ column) that did not pass the Wilcoxon rank sum test (** ***P-value >0.05*** **) between the ribosomal densities profiles when all genes aligned to the ORF's 5′ end (2^nd^ column) and 3′ end (3^rd^ column) of the genes from the** ***20%*** **top and bottom half-life.**
(PDF)

Table S5 **The functional genes groups of the three GO.** For each GO the names of the functional genes groups are listed, as well as the number of analyzed genes in each group out of the *1,525* highly translated genes.
(XLSX)

Table S6 **The lists of genes used in different parts of the study and the Wilcoxon comparison for various cut-offs and for mRNA HL in different conditions.**
(XLSX)
