# Supplementary figures and images for: Quantifying the Effect of Ribosomal Density on mRNA Stability
Source: PLoS One. 2014 Jul 14;9(7):e102308. doi: 10.1371/journal.pone.0102308 (PMC4096589; doi:10.1371/journal.pone.0102308)

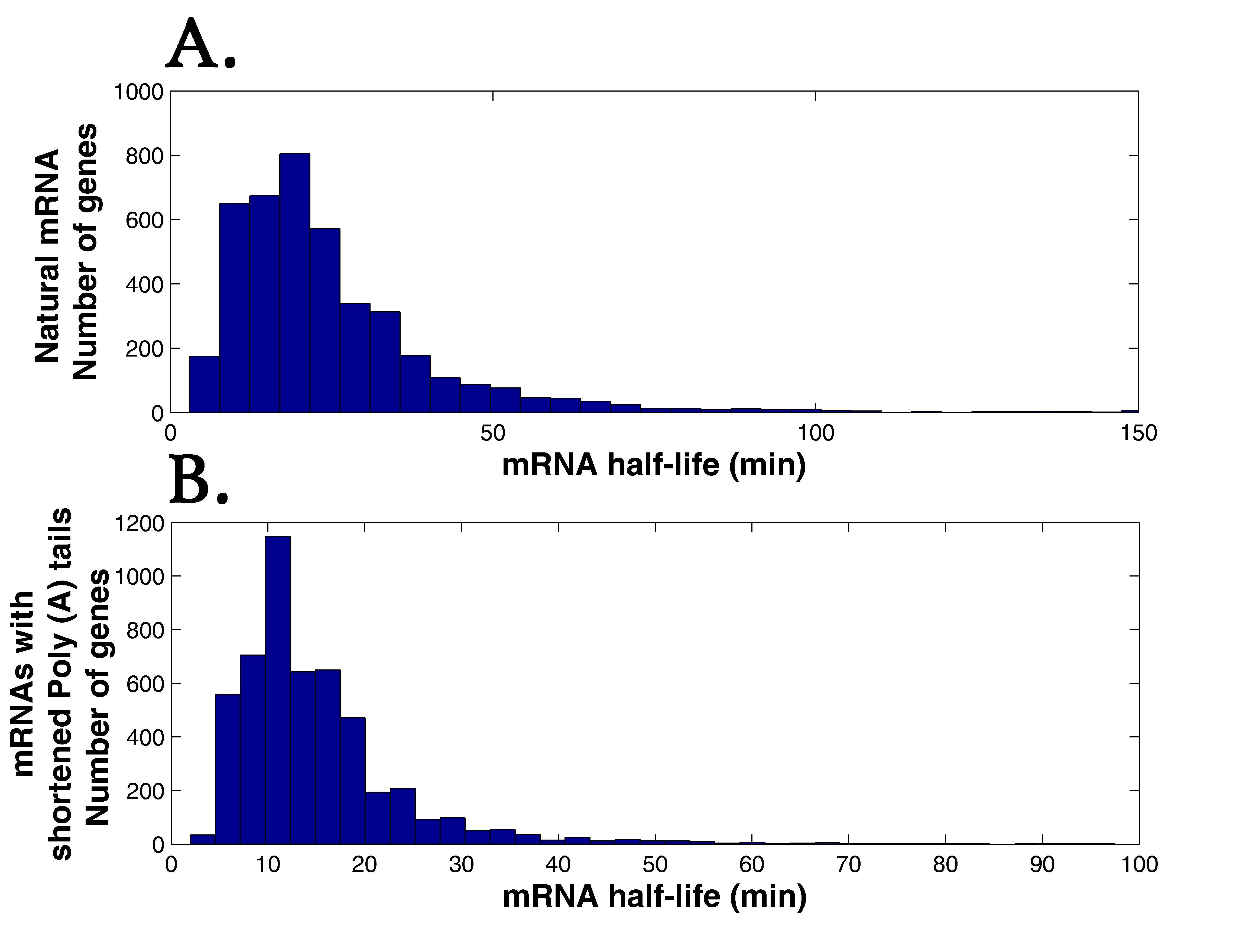

Supplement: Figure S1 — Histograms of the two half-life decay experiments from ref. [31] study: (A) for natural mRNA molecules and (B) for mRNAs with shortened Poly (A) tails. (JPG) [file pone.0102308.s001.jpg]

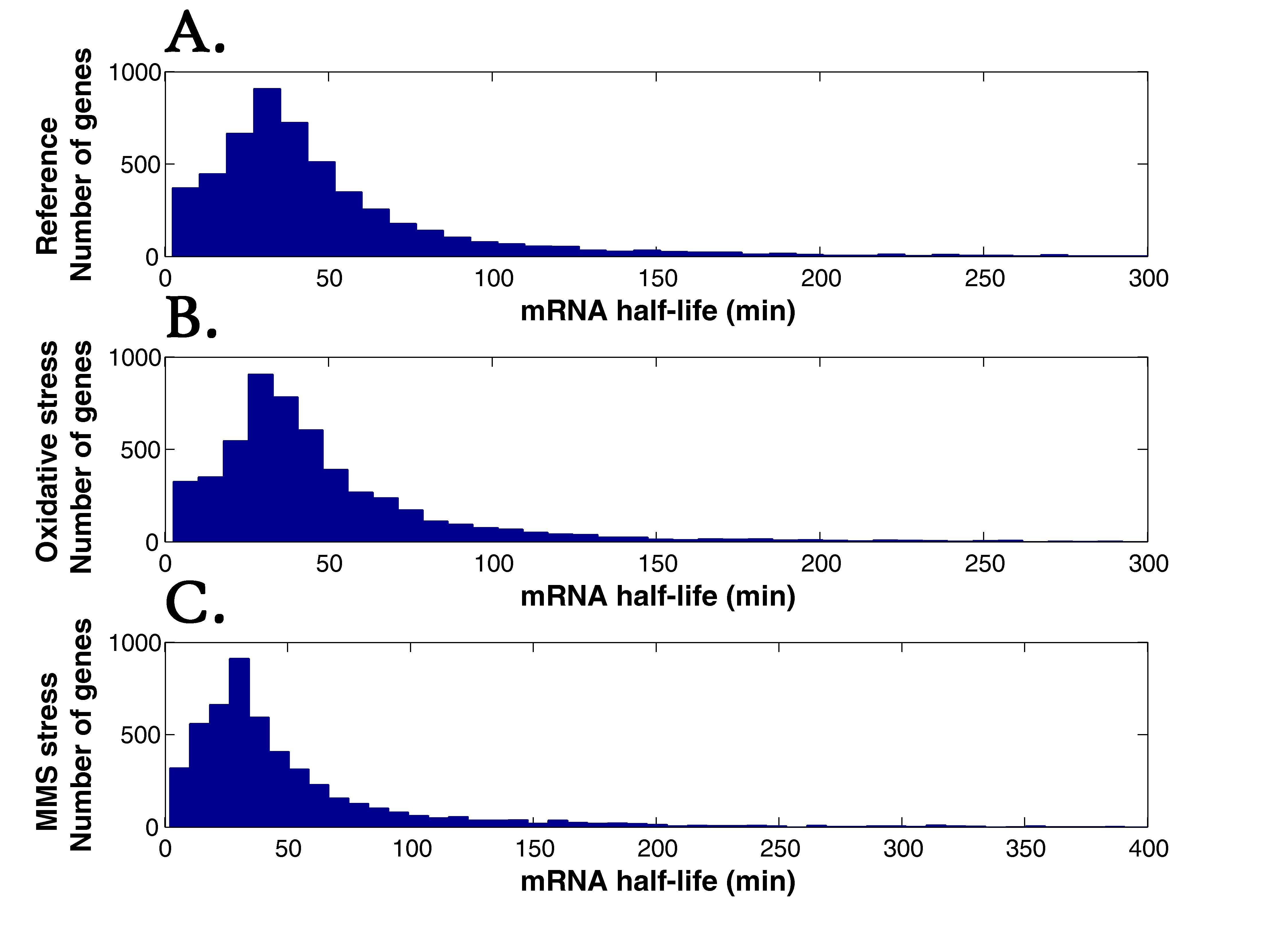

Supplement: Figure S2 — Histograms of the different half-life decay experiments data from ref. [6] study – (A) a reference experiment and two different environmental conditions: (B) exposure to oxidative stress and (C) exposure to MMS. (JPG) [file pone.0102308.s002.jpg]

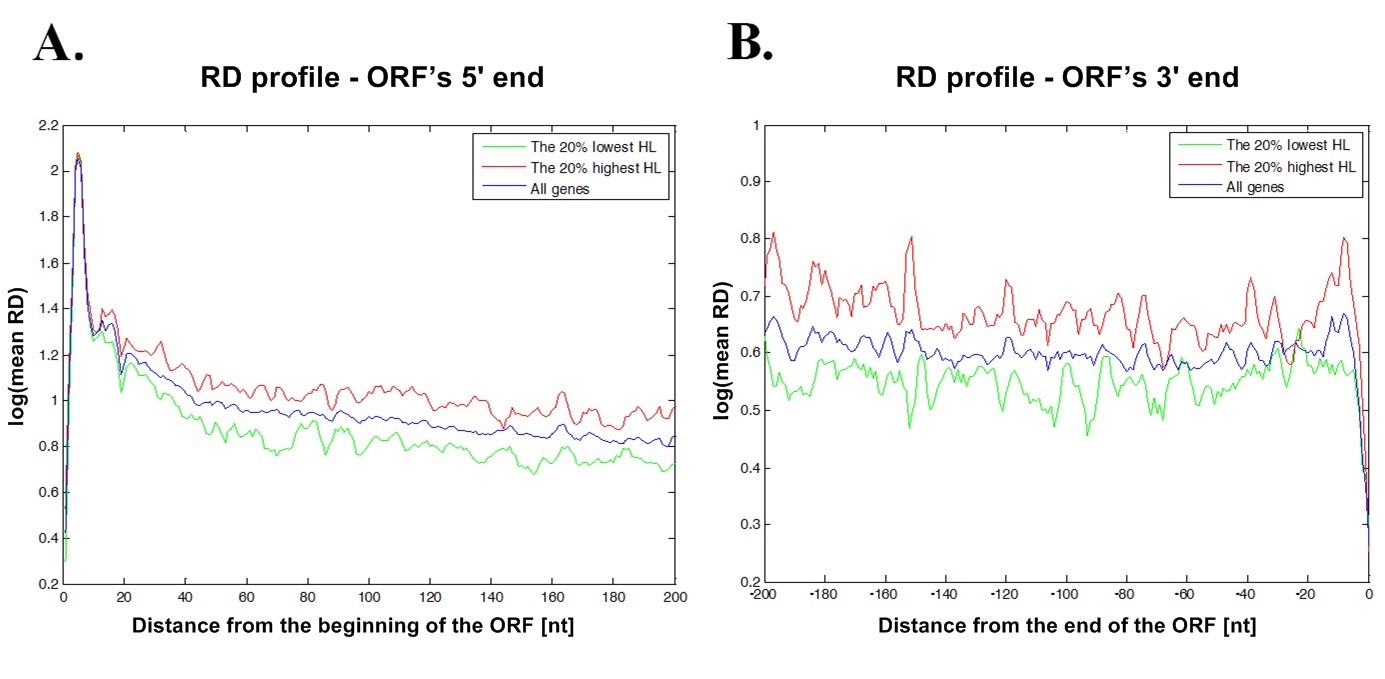

Supplement: Figure S3 — Ribosomal density (RD) profile for highly translated genes at single nucleotide resolution. (A) The first 200 nts; all genes are aligned to the ORF's 5′ end. (B) The last 200 nts; all genes are aligned to the ORF's 3′ end. The y-axes represents the mean RD in logarithmic scale at specific location along the ORF; the x-axes represents the location of a nucleotide measured as a distance from the ORF's 5′ end (positive numbers at (A)) or distance from the ORF's 3′ end (negative number at (B)). The red line represents the 20% of genes with the longest half-life; the green line represents the 20% of genes with the shortest half-life and the blue line represents all the genes. (JPG) [file pone.0102308.s003.jpg]

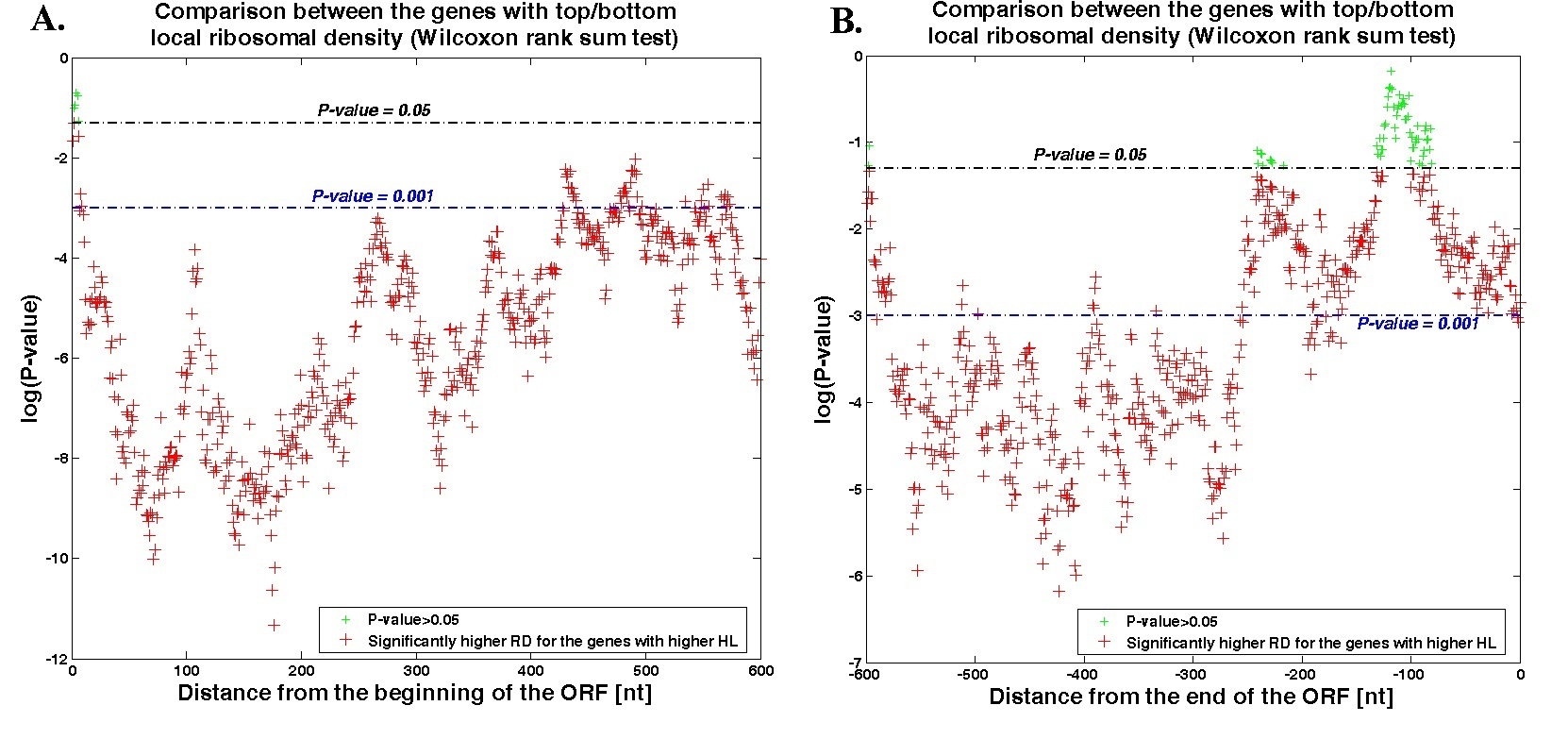

Supplement: Figure S4 — Wilcoxon rank sum test between mRNA half-lives of genes from the top and bottom 20% RD for each 40 nts sliding window at a resolution of single nt. The RD data were calculated as the average RD that were normalized with the mRNA levels averaged from refs. [29], [31], [32] data. (A) The ORF's first 600 nts; genes are aligned to the ORF's 5′ end. (B) The ORF's last 600 nts; genes are aligned to the ORF's 3′ end. The x-axis represents the location of the sliding window downstream to the aligned ORF's 5′ end (positive numbers at (A)) and upstream to the aligned ORF's 3′ end (negative number at (B)) respectively; the y-axis represents the log (Wilcoxon test P-value); the black line indicates that P-value = 0.05; the blue line indicates that P-value = 0.001. The green cross indicates that there is no significant difference between the half-life medians of the two groups; the red cross indicates that the half-life median of the genes group with higher RD is significantly higher than the one of the genes group with lower RD (P-value ≤0.05). There were no positions with significant signal in the opposite direction (i.e. genes with higher RD that have significantly lower HL). (JPG) [file pone.0102308.s004.jpg]

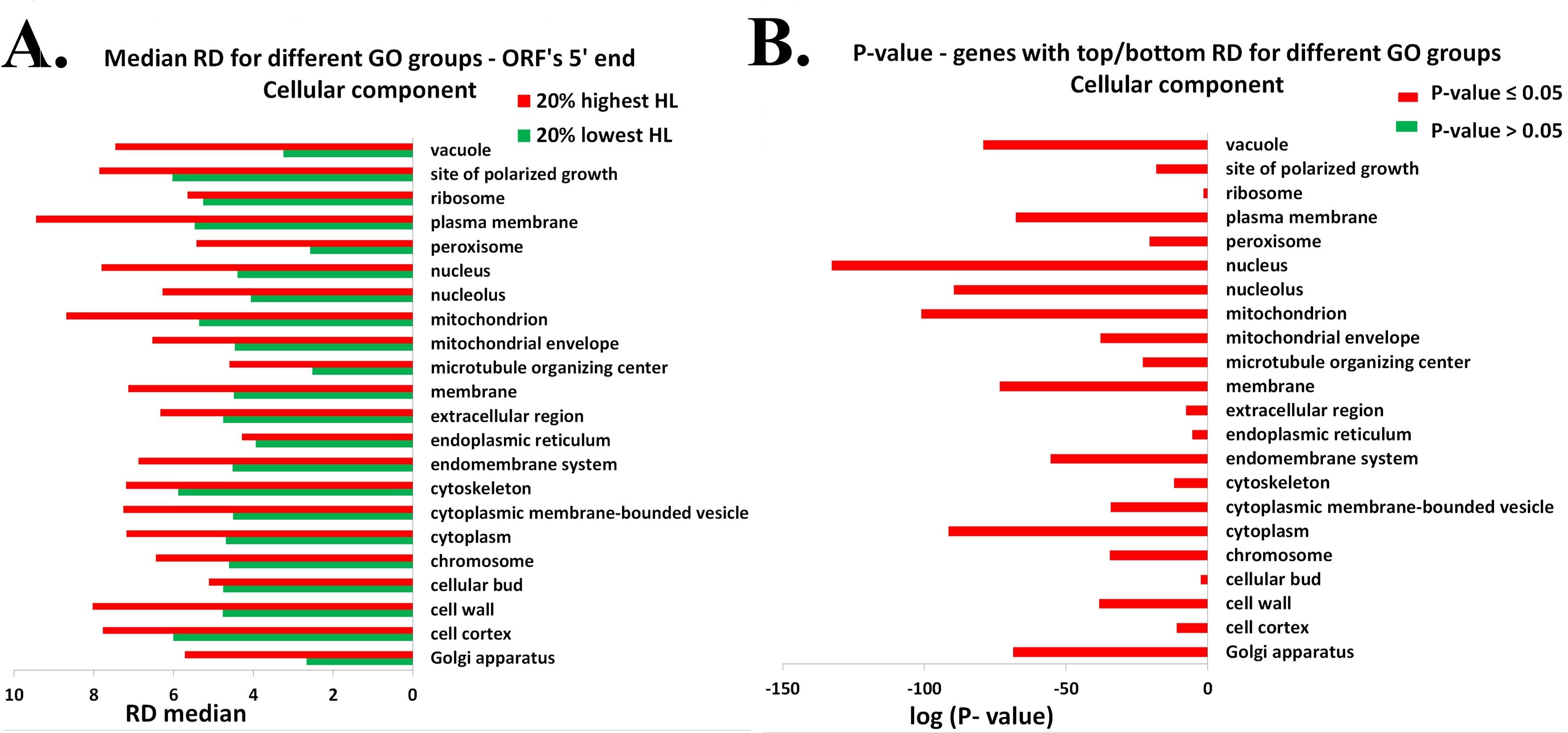

Supplement: Figure S5 — Cellular component GO: RD profile at single nucleotide resolution, of the first 600 nts when all genes are aligned to the ORF's 5′ end: (A) The RD median of the ORF's 5′ end RD profiles: the red/green bars represent the RD median of the 20% of the genes with top/bottom half-life. (B) Wilcoxon rank sum test between the RD profiles of the genes from the top and bottom 20% half-life for different functional genes groups. The x-axis represents the log (Wilcoxon test P-value) and the y-axis represents the functional genes group (see Table S5). Red bars indicate that P-value ≤0.05 whereas green bars indicate that P-value >0.05. (JPG) [file pone.0102308.s005.jpg]

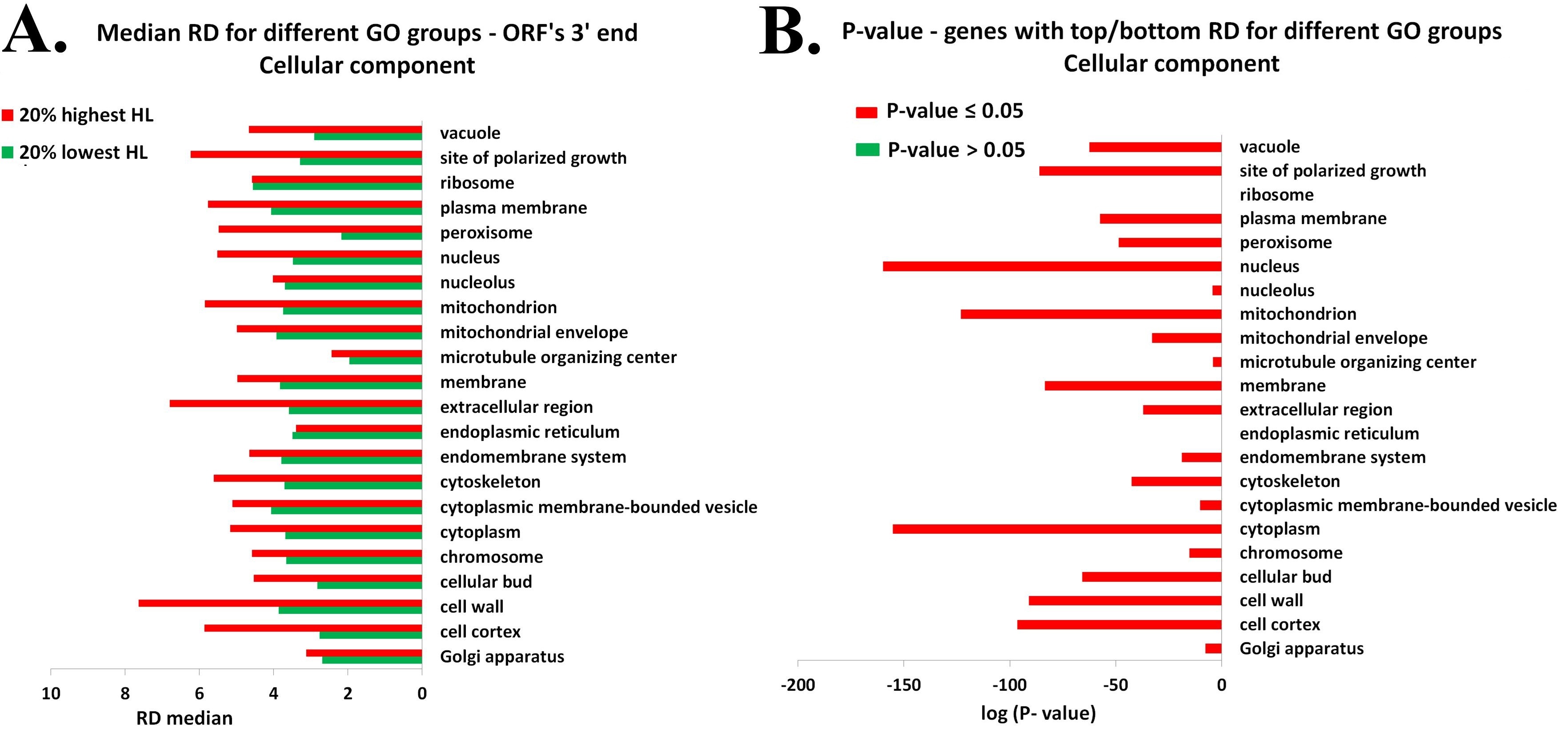

Supplement: Figure S6 — Cellular component GO: RD profile at single nucleotide resolution, of the last 600 nts when all genes are aligned to the ORFs 3′ end: (A) The RD median of the ORF's 3′ end RD profiles: the red/green bars represent the RD median of the 20% of the genes with top/bottom half-life. (B) Wilcoxon rank sum test between the ribosomal densities profiles of the genes from the top and bottom 20% half-life for different functional genes groups. The x-axis represents the log (Wilcoxon test P-value) and the y-axis represents the functional genes group (see Table S5). Red bars indicate that P-value ≤0.05 whereas green bars indicate that P-value >0.05. (JPG) [file pone.0102308.s006.jpg]

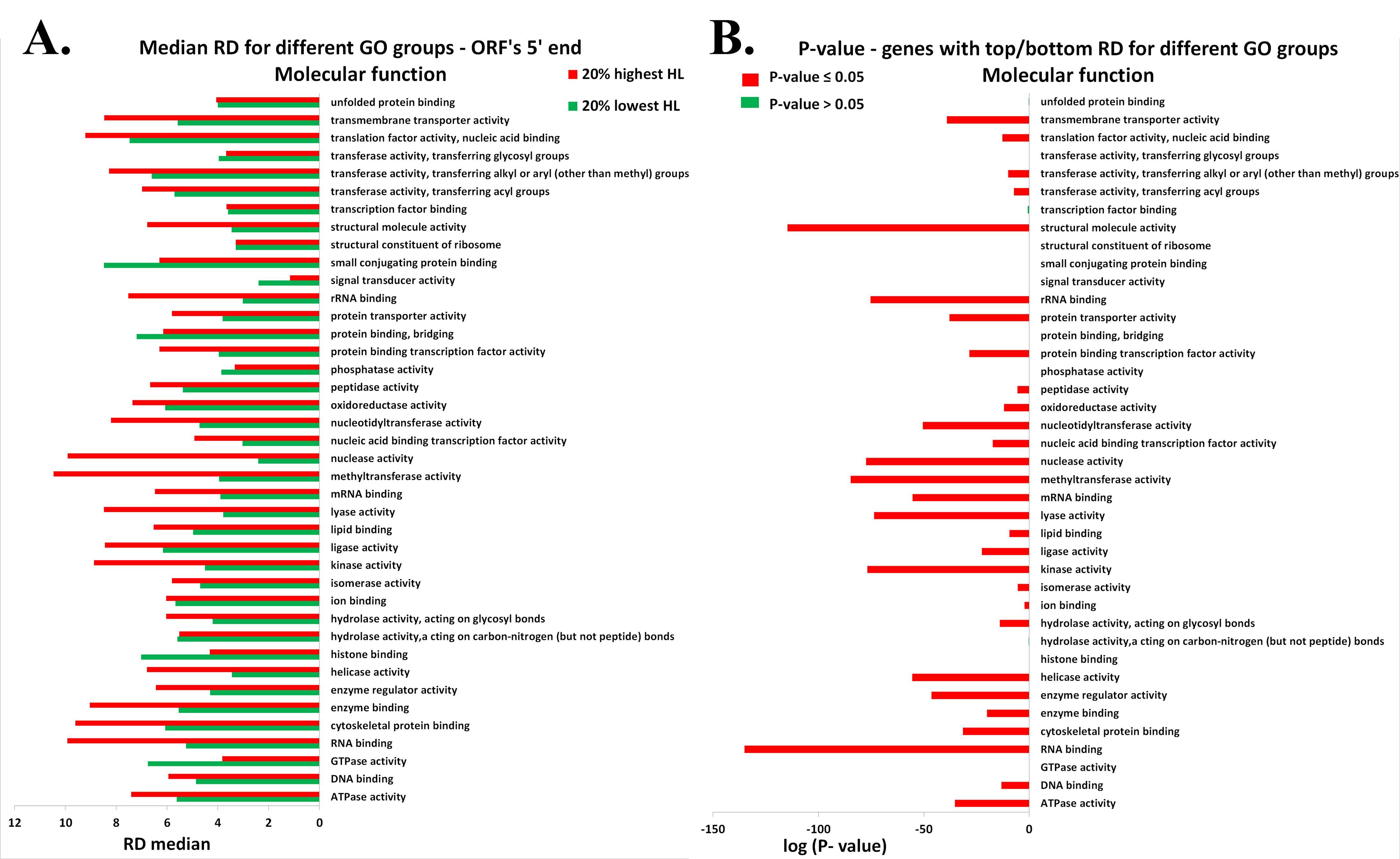

Supplement: Figure S7 — Molecular function GO: RD profile at single nucleotide resolution, of the first 600 nts when all genes are aligned to the ORF's 5′ end: (A) The RD median of the ORF's 5′ end RD profiles: the red/green bars represent the RD median of the 20% of the genes with top/bottom half-life. (B) Wilcoxon rank sum test between the RD profiles of the genes from the top and bottom 20% half-life for different functional genes groups. The x-axis represents the log (Wilcoxon test P-value) and the y-axis represents the functional genes group (see Table S5). Red bars indicate that P-value ≤0.05 whereas green bars indicate that P-value >0.05. (JPG) [file pone.0102308.s007.jpg]

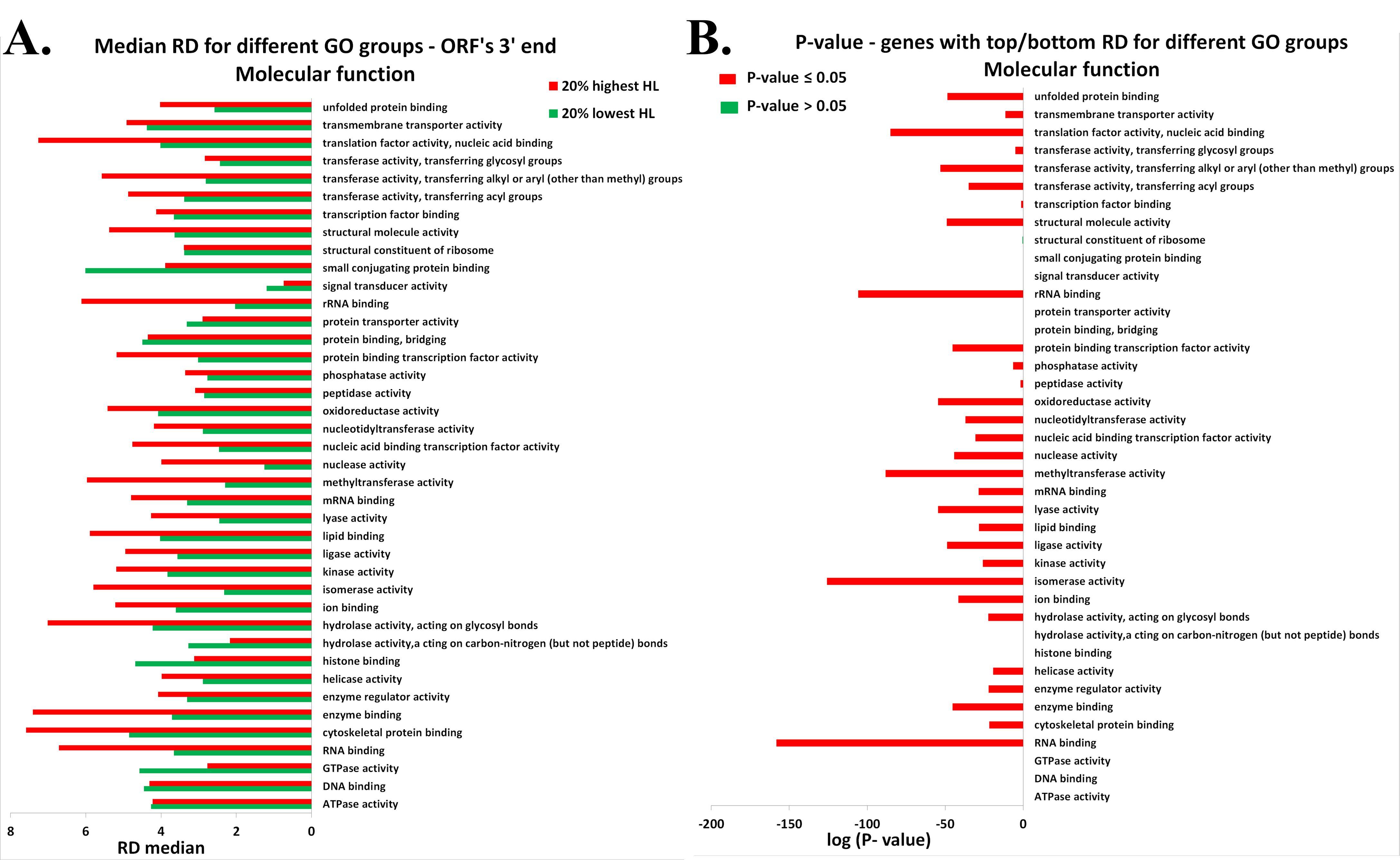

Supplement: Figure S8 — Molecular function GO: RD profile at single nucleotide resolution, of the last 600 nts when all genes are aligned to the ORFs 3′ end: (A) The RD median of the ORF's 3′ end RD profiles: the red/green bars represent the RD median of the 20% of the genes with top/bottom half-life. (B) Wilcoxon rank sum test between the ribosomal densities profiles of the genes from the top and bottom 20% half-life for different functional genes groups. The x-axis represents the log (Wilcoxon test P-value) and the y-axis represents the functional genes group (see Table S5). Red bars indicate that P-value ≤0.05 whereas green bars indicate that P-value >0.05. (JPG) [file pone.0102308.s008.jpg]

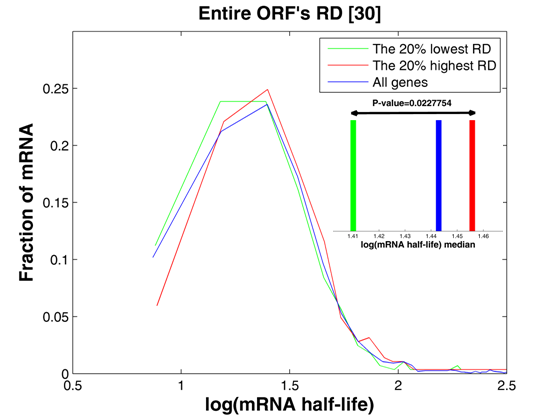

Supplement: Figure S9 — mRNA half-life distributions based on the data of Arava et al . Half-life distributions of the genes from the bottom 20% RD (green curve), top 20% RD (red curve), and of all genes (blue curve). The inset includes the median of each curve, which is represented by the intersection with the x-axis of a vertical line with the appropriate color: green, red and blue lines that indicate the half-life medians of the genes from the bottom and top 20% RD and of all genes respectively; for a better visualization, the graphs are based on the log (mRNA HL) values. The number above the arrow is the P-value corresponding to the Wilcoxon rank sum test between the mRNA HL of genes with the top and bottom 20% RD. (TIF) [file pone.0102308.s009.tif]

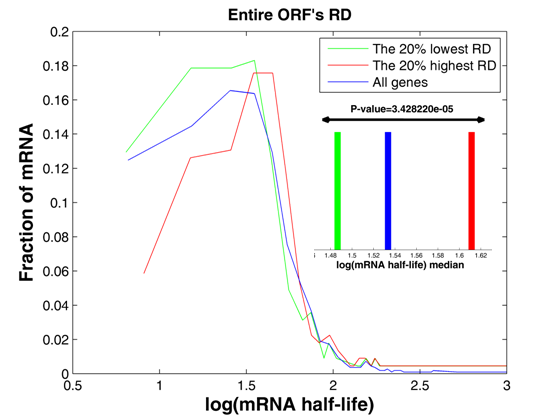

Supplement: Figure S10 — mRNA half-life distributions based genes that their ratio between the number of proteins and mRNA levels is larger than 100 . Half-life distributions of the genes from the bottom 20% RD (green curve), top 20% RD (red curve), and of all genes in the group (blue curve). The inset includes the median of each curve, which is represented by the intersection with the x-axis of a vertical line with the appropriate color: green, red and blue lines that indicate the half-life medians of the genes from the bottom and top 20% RD and of all genes in the group respectively; for a better visualization, the graphs are based on the log (mRNA HL) values. The number above the arrow is the P-value corresponding to the Wilcoxon rank sum test between the mRNA HL of genes with the top and bottom 20% RD. (TIF) [file pone.0102308.s010.tif]

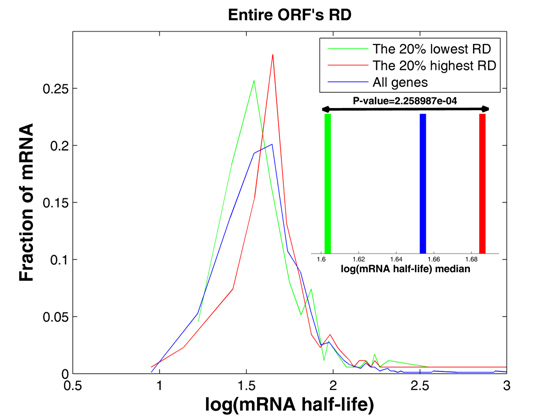

Supplement: Figure S11 — mRNA half-life distributions based genes that their ratio between mRNA half-life and the estimated translation time is larger than 50. Half-life distributions of the genes from the bottom 20% RD (green curve), top 20% RD (red curve), and of all genes in the group (blue curve). The inset includes the median of each curve, which is represented by the intersection with the x-axis of a vertical line with the appropriate color: green, red and blue lines that indicate the half-life medians of the genes from the bottom and top 20% RD and of all genes in the group respectively; for a better visualization, the graphs are based on the log (mRNA HL) values. The number above the arrow is the P-value corresponding to the Wilcoxon rank sum test between the mRNA HL of genes with the top and bottom 20% RD. (TIF) [file pone.0102308.s011.tif]
