# Supplementary material for: Quantifying the Effect of Ribosomal Density on mRNA Stability
Source: PLoS One. 2014 Jul 14;9(7):e102308. doi: 10.1371/journal.pone.0102308 (PMC4096589; doi:10.1371/journal.pone.0102308)
Supplement: Table S1 — Spearman correlation (and P-values) between the different half-life decay experiments data from ref. [6] . - a reference experiment and two different environmental conditions: exposure to oxidative stress and exposure to MMS. (PDF) [file pone.0102308.s012.pdf]

	Reference	Oxidative stress	MMS stress
**Reference**	*1*	*0.8014 (<10^{-08})*	*0.6292 (<10^{-09})*
**Oxidative stress**		*1*	*0.5497 (<10^{-09})*
**MMS stress**			*1*
