# Supplementary material for: Quantifying the Effect of Ribosomal Density on mRNA Stability
Source: PLoS One. 2014 Jul 14;9(7):e102308. doi: 10.1371/journal.pone.0102308 (PMC4096589; doi:10.1371/journal.pone.0102308)
Supplement: Table S2 — Spearman correlation (and P-values) between the datasets of the different half-life decay experiments from refs. [6], [31] . studies. (PDF) [file pone.0102308.s013.pdf]

	Reference	Oxidative stress	MMS stress	Natural mRNA	Shortened Poly(A) tail
**Reference**	*1*	*0.8014 (<10^{-08})*	*0.6292 (<10^{-09})*	*0.4240 (10^{-174})*	*0.4021 (10^{-185})*
**Oxidative stress**		*1*	*0.5497 (<10^{-09})*	*0.3762 (10^{-136})*	*0.3731 (10^{-159})*
**MMS stress**			*1*	*0.2861 (10^{-73})*	*0.3102 (10^{-103})*
**Natural mRNA**				*1*	*0.6226 (<10^{-323})*
**Shortened Poly(A) tail**					*1*
