# Supplementary material for: Quantifying the Effect of Ribosomal Density on mRNA Stability
Source: PLoS One. 2014 Jul 14;9(7):e102308. doi: 10.1371/journal.pone.0102308 (PMC4096589; doi:10.1371/journal.pone.0102308)
Supplement: Table S3 — Spearman correlation (and P-values) between the different mRNA levels datasets from refs. [29], [31], [32] . studies. (PDF) [file pone.0102308.s014.pdf]

	Brar, 2012	Ingolia, 2009	Wang, 2002
**Brar et al., 2012**	*1*	*0.6193 (<10^{-323})*	*0.5884 (<10^{-323})*
**Ingolia et al., 2009**		*1*	*0.7378 (<10^{-324})*
**Wang et al., 2002**			*1*
