# Supplementary material for: Quantifying the Effect of Ribosomal Density on mRNA Stability
Source: PLoS One. 2014 Jul 14;9(7):e102308. doi: 10.1371/journal.pone.0102308 (PMC4096589; doi:10.1371/journal.pone.0102308)
Supplement: Table S4 — The functional groups divided to the three GO domains (1st column) that did not pass the Wilcoxon rank sum test ( P-value >0.05 ) between the ribosomal densities profiles when all genes aligned to the ORF's 5′ end (2nd column) and 3′ end (3rd column) of the genes from the 20% top and bottom half-life. (PDF) [file pone.0102308.s015.pdf]

GO	RD ORF 5' end<br>*P-value > 0.05*	RD ORF 3' end<br>*P-value > 0.05*
**Cellular component**	None	• Endoplasmic reticulum<br>• Ribosome
**Molecular function**	• GTPase activity<br>• Histone binding<br>• Hydrolase activity, acting on carbon-nitrogen (but not peptide) bonds<br>• Phosphatase activity<br>• Protein binding, bridging<br>• Signal transducer activity<br>• Small conjugating protein binding<br>• Structural constituent of ribosome<br>• Transcription factor binding<br>• Transferase activity, transferring glycosyl groups<br>• Unfolded protein binding	• ATPase activity<br>• DNA binding<br>• GTPase activity<br>• Histone binding<br>• Hydrolase activity, acting on carbon-nitrogen (but not peptide) bonds<br>• Protein binding, bridging<br>• Protein transporter activity<br>• Signal transducer activity<br>• Small conjugating protein binding<br>• Structural constituent of ribosome
**Biological process**	• Golgi vesicle transport<br>• Carbohydrate transport<br>• Conjugation<br>• Endocytosis<br>• Meiotic cell cycle<br>• Membrane invagination<br>• Nucleus organization<br>• Organelle inheritance	• Golgi vesicle transport<br>• RNA modification<br>• Carbohydrate transport<br>• Cell budding<br>• Cellular ion homeostasis<br>• Cellular respiration<br>• Chromosome segregation<br>• Endocytosis

• Protein glycosylation<br>• Response to heat<br>• snoRNA processing<br>• sporulation<br>• tRNA processing<br>• Transcription from RNA polymerase III promoter<br>• Translational initiation<br>• Transposition<br>• Vitamin metabolic process	• exocytosis<br>• Generation of precursor metabolites and energy<br>• Lipid metabolic process<br>• Lipid transport<br>• Meiotic cell cycle<br>• Membrane invagination<br>• Mitochondrial translation<br>• Mitochondrion organization<br>• Nucleus organization<br>• Organelle inheritance<br>• Peroxisome organization<br>• Protein maturation<br>• Response to heat<br>• snoRNA processing<br>• tRNA processing<br>• Transcription from RNA polymerase I promoter<br>• Transcription from RNA polymerase III promoter<br>• Transposition
